# Label-free differentiation of classical and hypermobile Ehlers–Danlos syndromes using Mueller matrix polarimetry

**DOI:** 10.1117/1.BIOS.3.1.015002

**Published:** 2026-01-23

**Authors:** Kseniia Tumanova, Eric Min, Daniel C. Louie, Mehrnoosh Neshatian, Sara Shalviry, Nimish Mittal, Laurent Bozec, Alex Vitkin

**Affiliations:** a University of Toronto, Department of Medical Biophysics, Temerty Faculty of Medicine, Toronto, Ontario, Canada; b University Health Network, Princess Margaret Cancer Centre, Toronto, Ontario, Canada; c University of Toronto, Faculty of Dentistry, Toronto, Ontario, Canada; d University of Toronto, Division of Physical Medicine and Rehabilitation, Temerty Faculty of Medicine, Toronto, Ontario, Canada; e University of Toronto, Department of Radiation Oncology, Temerty Faculty of Medicine, Toronto, Ontario, Canada

**Keywords:** Ehlers–Danlos syndrome, biophotonics, Mueller matrix microscopy

## Abstract

**Background:**

Diagnosing classical and hypermobile Ehlers–Danlos syndromes, cEDS and hEDS, respectively, remains challenging due to overlapping clinical presentations and the lack of objective diagnostic tools. A label-free quantitative method for detecting EDS from normal tissues and for distinguishing its subtypes could support more timely and accurate clinical evaluation.

**Objectives:**

We evaluate the capability of Mueller matrix (MM) polarized light microscopy to resolve polarimetric contrasts between healthy and EDS samples and to assess its potential for differentiating between the cEDS and hEDS subtypes.

**Methods:**

Unstained skin biopsy slides from 19 participants (healthy, n=3; cEDS, n=5; hEDS, n=11) were imaged using a custom MM microscopy setup. In addition to acquiring 16 MM elements, 24 polarimetric parameters were obtained and tested for their detection and differentiation abilities. Group-wise comparisons of median parameter values were performed after assessing distribution normality using the Shapiro–Wilk test, applying either the Mann–Whitney U test or the independent-samples t-test as appropriate.

**Results:**

Three polarimetric parameters ($P_{L}$, β, and ψ) showed statistically significant differences between EDS-affected and healthy skin, with higher $P_{L}$ values observed in healthy tissue, consistent with more uniform collagen alignment in healthy subjects. Five parameters ($P_{L}$, $r_{L}$, $P_{1}$, $P_{3}$, and $P_{\mathrm{tms}}$) significantly differentiated cEDS from hEDS, with $r_{L}$ demonstrating the strongest separation. $P_{L}$ was the only parameter to show statistical significance for both tasks.

**Conclusions:**

MM polarimetry shows promise for label-free, quantitative assessment of skin collagen organization and offers potential for detecting EDS and differentiating its subtypes.

Statement of Discovery This work utilizes Mueller matrix polarimetry to quantitatively evaluate unstained skin biopsies from healthy individuals and patients with classical and hypermobile Ehlers–Danlos syndromes. The results show measurable differences in polarization biomarkers between these groups.

## 1 Introduction

Ehlers–Danlos syndromes (EDS) are a group of heritable connective tissue disorders that primarily affect the extracellular matrix (ECM), and are commonly associated with symptoms such as skin hyperextensibility, joint hypermobility, and tissue fragility.^1^ Among its subtypes, classical EDS (cEDS) and hypermobile EDS (hEDS) are among the most frequently encountered in clinical practice.^1^^–^^3^ Although cEDS is typically linked to pathogenic variants in COL5A1 and COL5A2 genes, encoding type V collagen,^3^ hEDS currently lacks a confirmed genetic marker and is diagnosed clinically based on patient symptoms and exclusion criteria.^2^^,^^3^ Diagnosis of both subtypes is often delayed, with patients undergoing extended workups involving Beighton scoring for joint hypermobility, systemic feature assessment, and family history reviews.^1^^,^^2^^,^^4^ These evaluations are subjective, may be inconclusive, and in the case of hEDS, can result in diagnostic delays exceeding 10 years.^4^^,^^5^ The absence of a definitive molecular or imaging biomarker highlights the need for an objective diagnostic method to support clinical assessment as more reliable identification of EDS could shorten the diagnostic process, improve consistency of care, and help avoid unnecessary testing.

As one of the pathological changes in EDS affects type I collagen fibrillogenesis, direct visualization and quantification of collagen structure in the dermis could become an avenue to aid diagnosis.^1^^,^^6^^,^^7^ Although conventional histopathology has been used in select cases, its diagnostic sensitivity is limited.^8^^,^^9^ Routine hematoxylin and eosin (H&E) staining is not optimal for assessing collagen, and even with collagen-specific stains such as Masson’s trichrome^10^ or picrosirius red,^11^ characteristic abnormalities are identified in only a minority of patients.^1^^,^^10^^,^^11^ Transmission electron microscopy (TEM) can detect structural collagen abnormalities such as fibril cross-sectional disorganization and irregular contours,^12^ but it is very expensive and has not typically been applied to assess spatial features along the fibril axis, such as D-banding periodicity.^13^ From a diagnostic perspective, TEM is labor-intensive, technically complex, and not widely accessible.^13^^,^^14^ Alternative approaches, including atomic force microscopy (AFM)^15^ and polarized light imaging,^16^ have demonstrated that both cEDS and hEDS are associated with collagen fibril thinning, misalignment, and mechanical weakening, further supporting the potential value of collagen microstructure as a measurable diagnostic feature.^1^^,^^17^ These studies confirm that structural collagen abnormalities are present in EDS dermis and necessitate systematic quantification to evaluate their potential diagnostic value.

Mueller matrix polarimetry (MMP) is a label-free optical imaging technique that characterizes tissue microstructure by quantifying how polarized light is modified upon transmission through thin tissue sections or reflection from bulk samples.^18^ Measurement of the complete 4×4 Mueller matrix enables derivation of parameters such as birefringence, depolarization, optical anisotropy, and dichroism, which may be directly related to tissue heterogeneity and collagen organization.^16^^,^^18^^–^^22^ In contrast to staining-based techniques, more complex laser-based methods, or nanomicroscopy, MMP offers a technologically and procedurally simple approach for efficient wide-field imaging of unstained tissue sections.^20^^,^^22^^,^^23^ Polarimetric parameters derived from MMP have been applied in various disease contexts involving changes in ECM architecture.^20^^,^^22^^–^^25^ In oncology, MMP has been used to identify differences in collagen structure associated with tumor progression, with polarimetric features correlating with clinical outcomes such as recurrence and survival.^20^^,^^22^^–^^26^

Given that cEDS and hEDS patients likely exhibit alterations in collagen alignment, abundance, and birefringence,^1^^–^^3^^,^^17^ MMP may offer a practical approach to identify these changes directly in skin tissue. The aim of the present study is thus to explore the ability of MMP to (1) differentiate between healthy skin and skin from patients with cEDS or hEDS and then (2) between these subtypes, by analyzing polarization-resolved features from unstained human skin biopsy sections. We hypothesize that polarimetric parameters extracted from these images can serve as structural biomarkers of disease, providing a quantitative and objective adjunct to existing diagnostic methods.

## 2 Materials and Methods

**Fig. 1:**
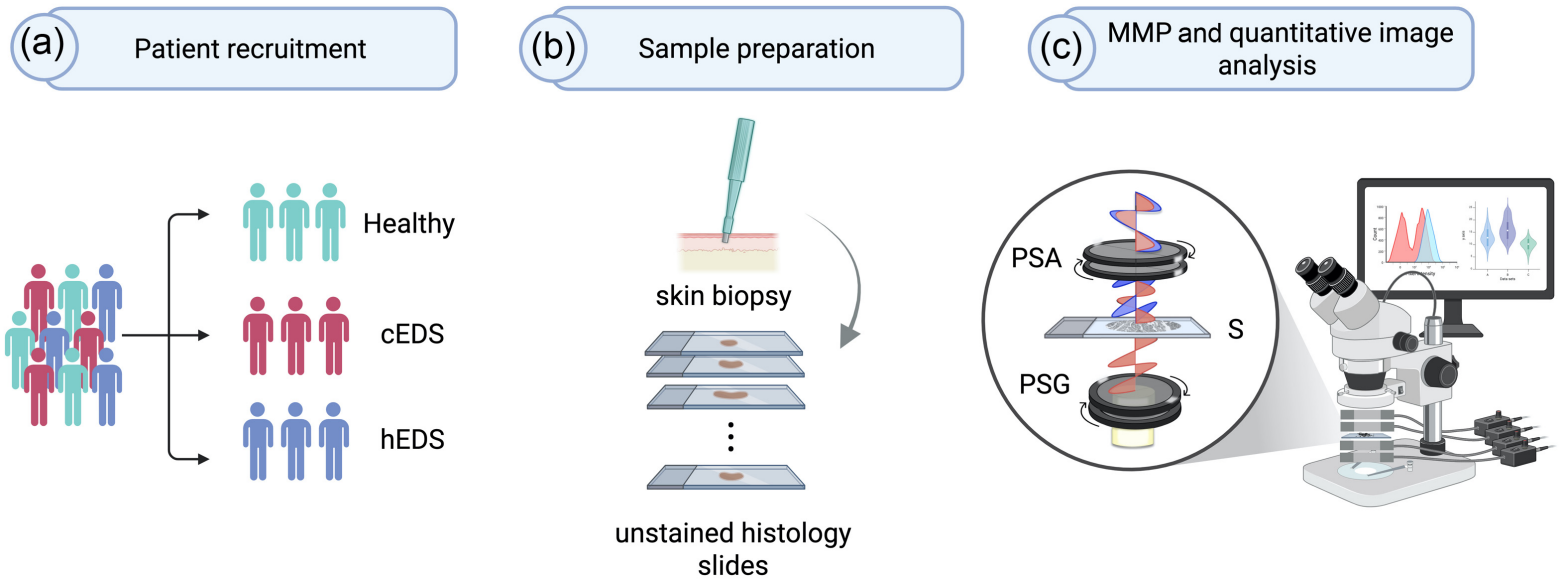
Experimental workflow for label-free polarimetric analysis of EDS skin biopsies. (a) Study participants were categorized into three groups: classical Ehlers–Danlos syndrome (cEDS), hypermobile Ehlers–Danlos syndrome (hEDS), and healthy controls; (b) skin punch biopsies were embedded in OCT, frozen, and sectioned at 7  μm; two serial sections were prepared per biopsy: one H&E-stained and one left unstained for MMP analysis; (c) unstained slides (S) were imaged using MMP. The system employed a polarization state generator (PSG) and a polarization state analyzer (PSA) to illuminate the sample and analyze the transmitted polarization states. The full 4×4 Mueller matrix was reconstructed based on a set of polarization-resolved transmission measurements. Analysis of extracted polarimetric parameters was then performed to assess differences among the groups.

Figure 1 illustrates the experimental workflow for MMP analysis of EDS skin biopsies, outlining the steps of tissue collection, simple sample preparation, polarimetric imaging, and data analysis. Detailed descriptions of each stage are provided below.

### 2.1 Tissue Samples

The study was approved by the University Health Network Research Ethics Board (UHN, Toronto, Canada; REB #21-5542). Written informed consent was obtained from all participants prior to study enrollment. The cohort included 19 individuals (mean age: 33±7 years), comprising 3 healthy controls, 5 diagnosed with cEDS, and 11 with hEDS. To be included in this study, cEDS cases required genetic confirmation by next-generation sequencing (NGS) with exon-targeted microarray analysis for copy number variants, performed at the Genome Diagnostics Laboratory, Hospital for Sick Children (Toronto, Canada). The gene panel interrogates 22 genes associated with connective tissue disorders.^27^ hEDS cases were included if they fulfilled the 2017 clinical diagnostic criteria.^28^ Healthy controls were individuals with no known connective tissue disorders. For each participant, a skin punch biopsy with a diameter of 3 mm was obtained from the medial surface of the upper arms above the elbow at the UHN GoodHope EDS Clinic. Biopsies were immediately placed in Dulbecco’s Modified Eagle Medium (DMEM; Sigma-Aldrich, St. Louis, Missouri, United States) containing 2% penicillin–streptomycin and 2% fungizone, kept on ice, and transported to the Faculty of Dentistry, University of Toronto.

All skin biopsies were embedded in optimal cutting temperature (OCT) compound (VWR, Richmond, Illinois, United States), frozen, and sectioned perpendicular to the skin surface at 7  μm thickness, extending through the epidermis and papillary dermis. For each sample, two serial sections were collected: one was stained with H&E for histology, and the other was left unstained for MMP analysis. H&E staining followed standard histological protocols.^1^ Slides for both stained and unstained samples were then mounted with coverslips using standard mounting media. For MMP, the entire biopsy section was imaged, without prior selection of regions of interest (ROIs) by a pathologist.

### 2.2 Polarimetric Image Acquisition

Whole-slide imaging was performed without any pathologist-assisted pre-selection of regions of interest (ROIs), thus minimizing potential subjective selection bias in the analysis. The polarized light microscopy (PLM) setup consisted of a series of optical components placed between the objective lens (Plan Neofluar Z 1.0x/0.25 NA, Oberkochen, Germany) and LED light source (Illuminator HXP 200C (D), Zeiss, Oberkochen, Germany) of a standard stereo zoom microscope (Axio Zoom V16, Zeiss), following the configuration of Tumanova et al.^20^ A 310 W white-light beam passed through a 630 nm bandpass filter (ET630/75 or ZET630/10, Chroma, Taoyuan City, Taiwan) before entering the polarization optics. The optics included a polarization state generator (PSG) and polarization state analyzer (PSA), with the sample placed between them (Fig. 1). The PSG comprised a rotatable linear polarizer (LPVISE100-A, Thorlabs, Newton, New Jersey, United States) and rotatable quarter-wave plate (AQWP05M-600, Thorlabs), generating input polarization states: linear (directions of 0 deg (H), 90 deg (V), 45 deg (P), or −45  deg (B)) and circular (right-handed (R) or left-handed (L) directions). The PSA included the same components in reverse order. The polarization optics were mounted in motorized angular rotation stages (PRM1/MZ8, Thorlabs), driven by electromechanical controllers (KDC101, Thorlabs) connected to a computer and operated via a LabVIEW program. A stand positioned the polarization optics over the translation stage, and a custom sample holder placed the sample between the PSG and PSA. Transmitted light was captured using the microscope’s camera (Hamamatsu ORCA-Flash4.0 V3 Digital CMOS camera, Hamamatsu, Japan), which had a 2048×2048  pixel array. Images were acquired at a pixel size of $1.625\times 1.625\;\mu m^{2}$ pixels, corresponding to a field of view of $3.33\times 3.33\;{\mathrm{mm}}^{2}$ (2048×2048  pixels). The optical system employed a 4× objective lens (NA = 0.25) with a 1× camera adapter. The experimentally validated resolution, determined using a 1951 USAF resolution target (R3L3SIN, Thorlabs), was <2.2  μm. The polarization state corresponding to each pixel was described by a four-component Stokes vector S, where I is total intensity, Q and U are intensity differences between orthogonal linear states, and V is the difference between circular states:^29^

$$\begin{array}{c} S=(\begin{array}{c} I\\ Q\\ U\\ V \end{array})=(\begin{array}{c} H+V\\ H-V\\ P-B\\ R-L \end{array}). \end{array}$$ (1)

Input Stokes vectors were measured without the sample; output Stokes vectors were obtained with the sample in place. Using linear algebra, input and output Stokes vectors were used to compute a 16-element Mueller matrix (MM) for each pixel [Eq. (2)]. Although 16 polarimetric images suffice to derive the MM, 24 images were acquired to improve robustness and increase signal-to-noise ratio (SNR).^23^^,^^30^

$$\begin{array}{c} S_{\text{out}\;}={\mathrm{MS}}_{\text{in}},\;M=(\begin{array}{cccc} m_{11} & m_{12} & m_{13} & m_{14}\\ m_{21} & m_{22} & m_{23} & m_{24}\\ m_{31} & m_{32} & m_{33} & m_{34}\\ m_{41} & m_{42} & m_{43} & m_{44} \end{array}). \end{array}$$ (2)

System calibration was performed using reference samples (air and retarders with known optical properties at various angles). Measured output Stokes vectors were compared to theoretical expectations, yielding calibration errors less than 1% to 5%, which was deemed acceptable for the requirements of this study.^31^ To correct artefacts caused by off-axis rays, such as slight rotational drift and polarization mismatch,^23^^,^^32^ images were co-registered in MATLAB 2024-b (Registration Estimator, SURF algorithm) prior to segmentation. To segment tissue regions from the background, a simple intensity-based mask was applied to the raw images. Pixels corresponding to areas of negligible transmitted light (i.e., slide background) were excluded, focusing analysis on tissue regions to improve SNR for subsequent polarimetric calculations.

### 2.3 Extraction of Polarimetric Information

The MM captures the full polarimetric response of the sample, but its 16 raw elements are not generally directly interpretable, and some are additionally sensitive to the in-plane orientation of the specimen.^33^^–^^35^ Several mathematical frameworks have been developed to reorganize the MM into more interpretable quantities. We selected four complementary approaches that yielded 24 derived parameters: (a) Lu-Chipman MM polar decomposition (MMPD),^36^ (b) MM transformation (MMT),^18^ (c) MM rotation invariance (MMRI),^37^^,^^38^ and (d) MM linear identity (MMLI),^39^ which capture distinct aspects of optical polarization behavior, including ordering, symmetry, anisotropy, heterogeneity, and deviations from idealized systems,^36^^–^^39^ and yield parameters that are both more interpretable and insensitive to in-plane sample orientation. The derived parameters can be interpreted as quantitative biomarkers of tissue polarization properties related primarily (not exclusively) to collagen architecture, which may aid in distinguishing EDS-affected skin from healthy controls and in characterizing differences between EDS subtypes.

#### 2.3.1 Lu–Chipman Mueller matrix polar decomposition (MMPD)

Using MMPD, MM is decomposed into depolarizer $M_{\Delta }$, retarder $M_{R}$, and diattenuator $M_{D}$ matrices, from which three core parameters are obtained [Eqs. (3a)–(3c)]: depolarization power Δ, which may reflect local structural disorder related to tissue heterogeneity;^16^^,^^40^ retardance R, describing phase shifts from birefringence possibly influenced by collagen density and orientation;^16^^,^^19^ and diattenuation D, indicating preferential absorption of polarization states^16^^,^^41^ and sensitivity to tissue anisotropy.^16^^,^^19^ R can be further split into linear δ and circular ψ contributions [Eqs. (4a) and (4b)],^16^ with ψ additionally sensitive to optically active biomolecules such as glucose or other chiral macromolecular structures.^42^^,^^43^

$$R=\cos^{-1}[\frac{tr(M_{R})}{2}-1],$$ (3a)

$$D=\sqrt{(m_{12}{)}^{2}+(m_{13}{)}^{2}+(m_{14}{)}^{2}}\in [0,1],$$ (3b)

$$\Delta =1-\frac{\left|\mathrm{tr}(M_{\Delta })-1\right|}{3}\in [0,1],$$ (3c)

$$\delta =\cos^{-1}\{[(M_{R}(2,2)+M_{R}(3,3){)}^{2}+(M_{R}(3,2)+M_{R}(2,3){)}^{2}{]}^{\frac{1}{2}}-1\}\in [0,\pi ],$$ (4a)

$$\psi =\frac{1}{2}\;\tan^{-1}[\frac{M_{R}(3,2)-M_{R}(2,3)}{M_{R}(2,2)-M_{R}(3,3)}]\in [-\frac{\pi }{4},\frac{\pi }{4}].$$ (4b)

### 2.3.2 Mueller matrix transformation (MMT)

MMT technique derives parameters [Eqs. (5a)–(5d)] directly from the upper-left 3×3 MM submatrix by fitting to trigonometric functions in polar coordinates.^18^^,^^40^ The resulting parameters are b, another measure of depolarization,^19^^,^^38^ $t_{1}$ and its normalized form A, which quantify anisotropy^19^^,^^38^ and β, which provides a measure of optical rotation (circular birefringence) and is also sensitive to the coexistence of multiple anisotropic effects within the tissue.^16^^,^^18^^,^^41^

$$t_{1}=\frac{\sqrt{(m_{22}-m_{33}{)}^{2}+(m_{23}+m_{32}{)}^{2}}}{2}\in [0,1],$$ (5a)

$$b=\frac{m_{22}+m_{33}}{2}\in [0,1],$$ (5b)

$$\beta =\frac{\left|m_{23}-m_{32}\right|}{2}\in [0,1],$$ (5c)

$$A=\frac{2b\cdot t_{1}}{b^{2}+t_{1}^{2}}\in [0,1].$$ (5d)

### 2.3.3 Mueller matrix rotation invariance (MMRI)

Building on this, MMRI focuses on azimuth rotation-invariant combinations [Eqs. (6a)–(6g)] of the MM “corners” and “edges,”^37^^,^^38^^,^^41^ including $r_{L}$ and $q_{L}$, which describe the interconversion of circular and linear polarization states^38^^,^^41^ and linear/circular polarizance $\left(P_{L},P_{C}\right)$, indicating preferential transmission of linear and circularly polarized light, respectively.^37^^,^^38^^,^^41^

$$P_{L}=\sqrt{(m_{21}{)}^{2}+(m_{31}{)}^{2}}\in [0,1],$$ (6a)

$$D_{L}=\sqrt{(m_{12}{)}^{2}+(m_{13}{)}^{2}}\in [0,1],$$ (6b)

$$P_{C}=m_{41}\in [-1,1],$$ (6c)

$$D_{C}=m_{14}\in [-1,1],$$ (6d)

$$q_{L}=\sqrt{(m_{42}{)}^{2}+(m_{43}{)}^{2}}\in [0,1],$$ (6e)

$$r_{L}=\sqrt{(m_{24}{)}^{2}+(m_{34}{)}^{2}}\in [0,1],$$ (6f)

$$k_{C}=m_{44}\in [-1,1].$$ (6g)

Changes in collagen abundance and microstructure or disruptions in fiber organization associated with disease progression, such as in fibrosis and cancer, may influence these values, potentially allowing these parameters to reflect pathological alterations.^20^^,^^21^^,^^24^^,^^44^

### 2.3.4 Mueller matrix linear identity (MMLI)

MMLI provides a complementary set of parameters [Eqs. (7a)–(7i)] that quantify deviations from idealized systems,^39^ which may be useful for biological tissues that rarely exhibit idealistic optical behavior.^45^

$$P_{1}=\frac{m_{43}+m_{34}}{2}\in [-1,1],$$ (7a)

$$P_{2}=\frac{m_{42}+m_{24}}{2}\in [-1,1],$$ (7b)

$$P_{3}=|q_{L}-r_{L}|\in [-1,1],$$ (7c)

$$P_{4}=\frac{m_{34}\cdot m_{24}-m_{42}\cdot m_{43}}{2}\in [-1,1],$$ (7d)

$$P_{5}=\frac{m_{12}-m_{21}}{2}\in [-1,1],$$ (7e)

$$P_{6}=\frac{m_{13}-m_{31}}{3}\in [-1,1],$$ (7f)

$$P_{7}=|D_{L}-P_{L}|\in [-1,1],$$ (7g)

$$P_{8}=\frac{m_{12}\cdot m_{13}-m_{21}\cdot m_{31}}{2}\in [-1,1],$$ (7h)

$$P_{\mathrm{tms}}=\sqrt{P_{1}^{2}+P_{2}^{2}}\in [0,\sqrt{2}],$$ (7i)

Parameters $P_{1}$ to $P_{4}$ and $P_{5}$ to $P_{8}$ mark departures from pure linear retarders and diattenuators, respectively,^39^ whereas $P_{\mathrm{tms}}$ captures deviations from mirror-transverse symmetry, which is an invariant to illumination from opposite sides.^39^^,^^46^ For thin tissue sections, this corresponds to symmetry across the transverse plane of the sample, perpendicular to the direction of light propagation.^38^^,^^46^

Given the limited understanding of how EDS-related alterations in the dermal collagen organization affect the polarization of light, we applied all four approaches to comprehensively evaluate which biomarkers are most sensitive to variations in collagen architecture across control, cEDS, and hEDS groups. Definitions, notation, and biophysical relevance of each parameter are summarized in Table 1.

**Table 1:** Summary of all polarimetric parameters investigated in this study with definitions and biophysical interpretations from the literature.

Parameter		Equation	Definition	Tentative biophysical meaning
1	Δ	(3c)	Depolarization power	Structural heterogeneity of tissue
2	b	(5b)	Linear depolarization power	
3	$P_{L}$	(6a)	Linear polarizance	Directional heterogeneity of birefringent fibers
4	$P_{C}$	(6c)	Circular polarizance	
5	D	(3b)	Diattenuation	
6	$D_{C}$	(6d)	Circular diattenuation	
7	$D_{L}$	(6b)	Linear diattenuation	
8	$t_{1}$	(5a)	Universal anisotropy degree	
9	A	(5d)	Normalized anisotropy	
10	$P_{5}$	(7e)	Deviation from pure linear diattenuator	
11	$P_{6}$	(7f)		
12	$P_{7}$	(7g)		
13	$P_{8}$	(7h)		
14	R	(3a)	Retardance	Density and alignment of birefringent fibrous structures
15	δ	(4a)	Linear retardance	
16	$q_{L}$	(6e)	Capability to transform linear to circular polarization	
17	$r_{L}$	(6f)	Capability to transform circular to linear polarization	
18	$P_{1}$	(7a)	Deviation from pure linear retarder	
19	$P_{2}$	(7b)		
20	$P_{3}$	(7c)		
21	$P_{4}$	(7d)		
22	$P_{\mathrm{tms}}$	(7i)	Deviation from mirror transversal symmetric system	Transverse asymmetry of fiber arrangement
23	ψ	(4b)	Circular retardance	Presence of chiral molecules
24	β	(5c)	Circular birefringence	Presence of chiral molecules/heterogeneity in fibril alignment

### 2.4 Quantitative Image Analysis and Statistical Testing

To quantitatively assess group-level differences in polarimetric biomarkers, each parameter yielded on the order of $10^{6}\;\text{pixel}$ values per slide within the masked tissue region. From these tissue-only distributions, the mean, median, and standard deviation were computed as statistical descriptors for each sample. Unless otherwise specified, the reported values refer to the per-sample medians, resulting in one value per patient. This summary-statistic approach was used to obtain a consistent per-sample descriptor and to avoid drawing conclusions from distributional features that cannot be reliably evaluated in a small, unbalanced cohort. Comparisons were conducted in two stages: (1) pooled EDS samples (classical and hypermobile subtypes) versus healthy controls, and (2) classical versus hypermobile EDS. Normality of median distributions was evaluated using the Shapiro–Wilk test.^47^ When normality was satisfied, group differences were evaluated using the independent-samples t-test^48^; otherwise, the Mann–Whitney U test was applied.^49^ A two-tailed significance threshold of p<0.05 was used throughout. Because the healthy control group was small, statistical results involving this group have limited reliability. These comparisons were thus included to provide initial contextual reference for the EDS subgroups, rather than to draw strong conclusions for the healthy versus EDS cohorts differentiation.

No samples were excluded. Although statistical outlier removal can enhance power and robustness, the small cohort size and absence of objective clinical justification (e.g., no association with age, diagnosis, or technical error) precluded such filtering. All data points were retained to ensure a comprehensive and unbiased evaluation of potential inter-patient variability and to preserve the integrity of real-world clinical heterogeneity in the dataset.

## 3 Results and Discussion

**Fig. 2:**
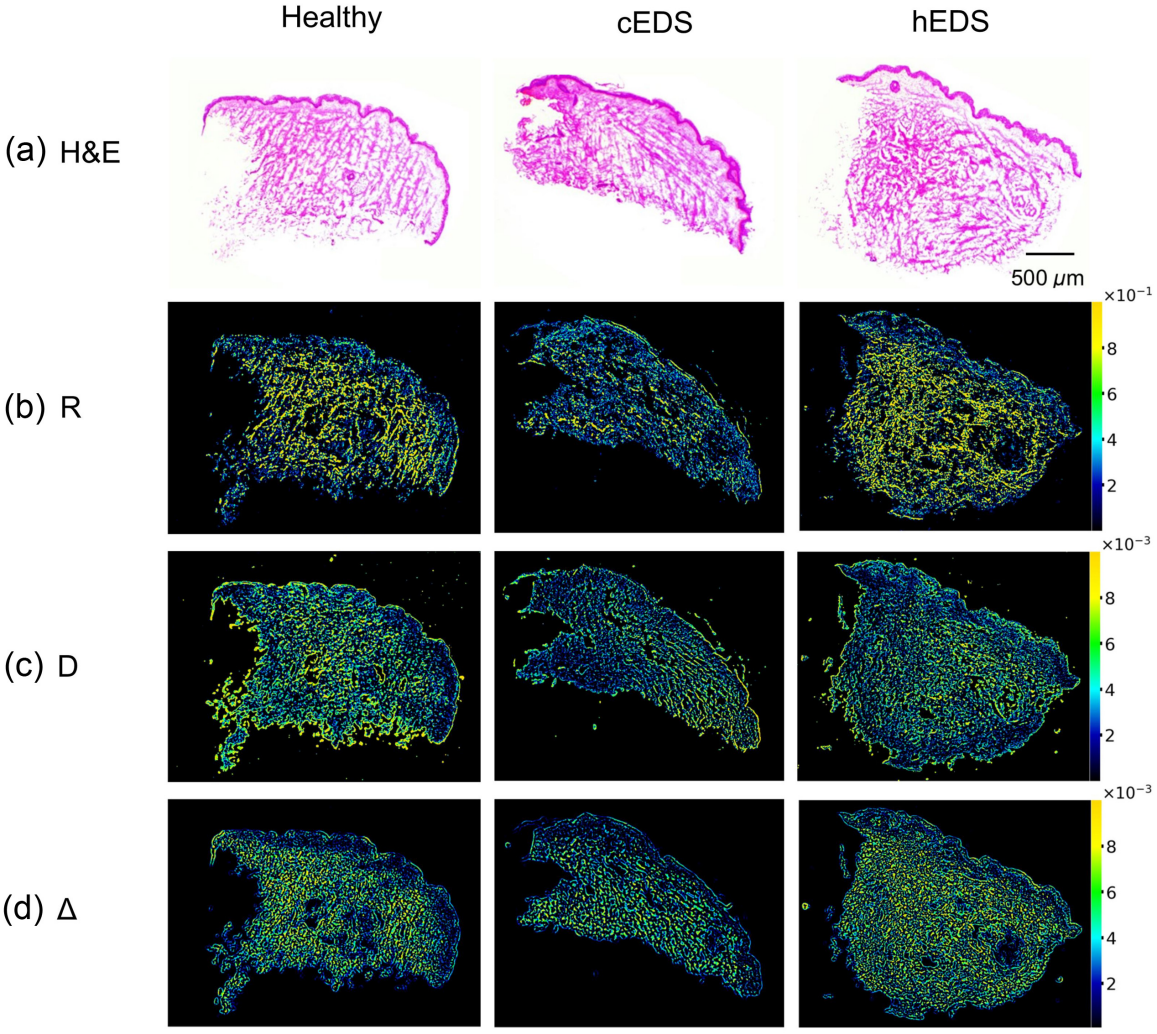
Representative (a) H&E-stained and (b)–(d) unstained skin biopsy sections from healthy controls, classical Ehlers–Danlos syndrome (cEDS), and hypermobile Ehlers–Danlos syndrome (hEDS) cases (left to right), imaged using brightfield whole-slide and polarized light microscopy (PLM). (a) H&E-stained sections. (b)–(d) Whole-slide Mueller matrix-derived maps of selected polarization parameters: (b) retardance (R), (c) diattenuation (D), and (d) depolarization power (Δ). Images are shown at the same magnification; the 500  μm scale bar in panel (a) applies to all panels. Visual differentiation across diagnostic groups remains challenging due to tissue heterogeneity and subtle contrast between healthy and diseased states, motivating subsequent statistical analysis of the polarimetric biomarkers; see text for details.

Representative images from healthy, cEDS, and hEDS skin biopsies are shown in Fig. 2.

In healthy controls, collagen bundles appear thick and well-organized, whereas EDS tissues show thinner, disordered fibers, and more heterogeneous orientation.^1^ Although H&E staining is the gold standard for histological evaluation, it has limited sensitivity for subtle differences in collagen alignment and architectural organization in EDS.^1^ In Figs. 2(b)–2(d), representative maps of three commonly used MM polarimetric parameters in biomedical optics are shown: retardance (R) [Fig. 2(b)], diattenuation (D) [Fig. 2(c)], and depolarization power (Δ) [Fig. 2(d)], each providing distinct polarization contrast.^36^^,^^50^^,^^51^ However, visual assessment of these polarimetric biomarkers did not reveal consistent group-level differences, necessitating quantitative analysis.

**Fig. 3:**
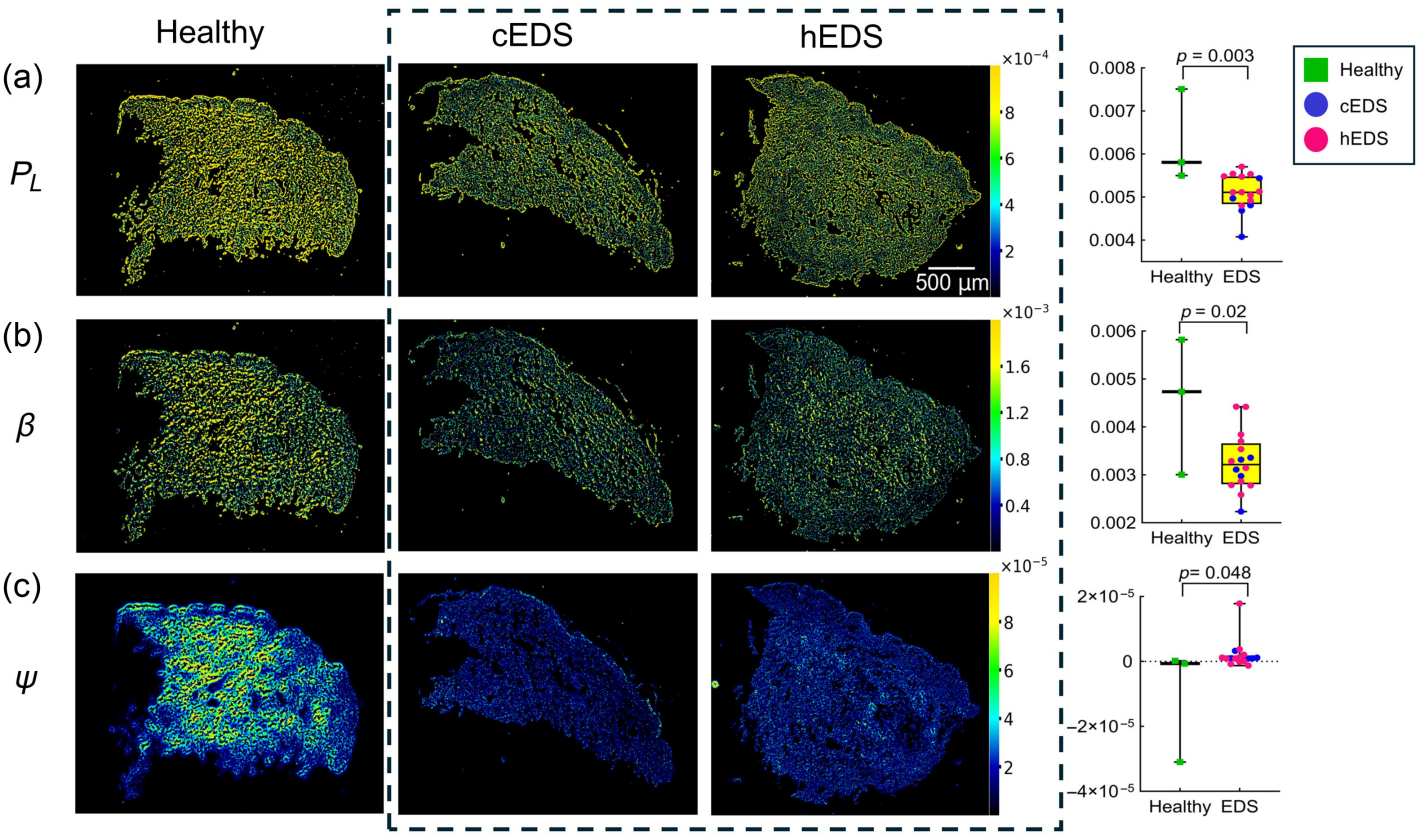
MMP images and corresponding box-and-whisker plots for (a) $P_{L}$, (b) β, and (c) ψ, the parameters that showed statistically significant (p<0.05) difference between healthy and EDS (cEDS and hEDS) skin biopsies (left to right). For each plot on the right, the central black line denotes the median, the box represents the interquartile range, and whiskers indicate the minimum and maximum values. Individual data points correspond to median pixel values from the full sample region. No outliers were excluded from the analysis (see Sec. 2.4). Statistical tests and p-values are shown in Table S1 in the Supplementary Material. Both EDS subtypes exhibited visibly lower pixel intensities for all three polarimetric biomarkers compared to healthy controls.

Among the MMP parameters assessed, $P_{L}$, β, and ψ differed significantly between healthy and EDS-affected biopsies. The representative $P_{L}$ image for the healthy tissues showed overall greater pixel intensities compared with cEDS and hEDS, reflecting greater values of this parameter (Fig. 3). This visual difference was supported by a two-tailed t-test (p=0.0033), as shown in Fig. 3 and detailed in Table S1 in the Supplementary Material. The higher $P_{L}$ values in healthy tissue suggest a preferential transmission of linearly polarized light, which may be attributed to more uniformly aligned collagen fibers, an organization known to be disrupted in EDS-affected tissues.^1^^,^^13^^,^^14^^,^^52^

A similar trend was observed for β, where healthy tissue exhibited greater values relative to EDS samples (p=0.02), suggesting a reduction in anisotropic effects associated with EDS-related structural alterations.^51^ Although ψ also demonstrated a statistically significant difference (p=0.048), most values in both groups clustered near zero. Two measurements deviated from this pattern, with one slightly below and one slightly above zero, contributing to the apparent group separation. This shift in sign may reflect subtle differences in collagen alignment, but given the small sample size, particularly in the healthy group (n=3), it is not possible to determine whether this represents a reproducible biological effect or sampling variability. The corresponding polarimetric images are shown in Fig. 3. Notably, no statistically significant differences were observed for total retardance R or linear retardance δ, consistent with the fact that total retardance is dominated by the linear component^36^ (Table S2 in the Supplementary Material). By contrast, both the circular retardance component ψ and β, which is also sensitive to circular birefringence, showed statistically significant differences between healthy and EDS skin (Fig. 3). These somewhat surprising findings suggest that circular birefringence may provide a useful source of contrast for distinguishing EDS from healthy tissue.

**Fig. 4:**
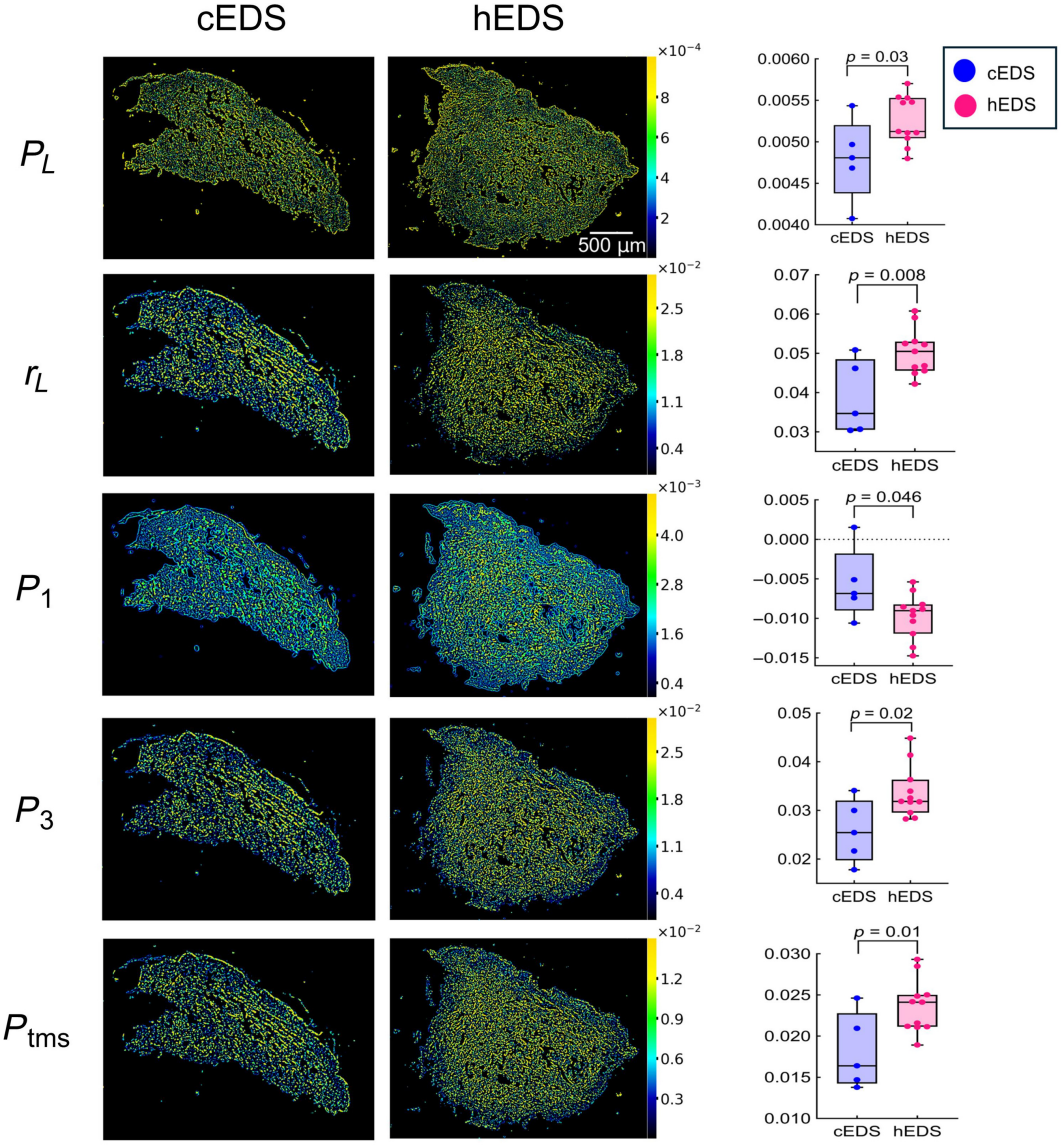
Representative MMP images and corresponding box-and-whisker plots for $P_{L}$, $r_{L}$, $P_{1}$, $P_{3}$, and $P_{\mathrm{tms}}$, the parameters that showed statistically significant differences (p<0.05) between cEDS and hEDS skin biopsies. In each plot on the right, the central black line indicates the median, the box represents the interquartile range, and whiskers denote the minimum and maximum values. Individual data points correspond to median pixel values from the full sample region. No outliers were excluded (see Statistical Analysis section). Statistical test and p-values are shown in Table S1 in the Supplementary Material.

In the comparison between EDS subtypes, five polarimetric biomarkers ($P_{L}$, $r_{L}$, $P_{1}$, $P_{3}$, $P_{\mathrm{tms}}$) showed statistically significant discriminating ability (Fig. 4). Among these parameters, $P_{L}$ remained statistically significant (p=0.03), though its discriminative strength was lower than in the healthy versus EDS comparison. $r_{L}$ demonstrated stronger statistical significance (p=0.008). Both parameters are associated with the linear polarization characteristics of the transmitted light.^37^^,^^38^

Their higher values in hEDS compared with cEDS may indicate differences in microstructural organization affecting polarization behavior.^1^^,^^38^ However, the interpretation of these metrics remains nontrivial, as $P_{L}$ and $r_{L}$ are derived from decomposition methods that capture complex polarization effects without directly isolating the underlying physical mechanisms.^37^^,^^38^ Similarly, $P_{1}$ (p=0.046), $P_{3}$ (p=0.02), and $P_{\mathrm{tms}}$ (p=0.01) values were greater in hEDS compared with cEDS samples. Theoretically, these parameters may be indicative of enhanced contributions from nonretardance-related effects ($P_{1}$, $P_{3}$) and/or asymmetries in polarization responses orthogonal to the direction of light propagation ($P_{\mathrm{tms}}$). Nonetheless, their interpretation is limited as they are computed from algebraic combinations of MM elements [Eqs. (8a) and (8c)], each lacking a clear direct physical meaning.^39^ As such, although statistically significant, these differences cannot yet be readily linked to specific tissue properties.

Overall, these observations suggest that, contrary to prior assumptions, fiber architecture in hEDS tissue may not necessarily be more disordered than in cEDS.^1^ In fact, the consistently higher values of linear polarization-related parameters in hEDS could indicate a relatively more organized microstructure with distinct anisotropies influencing polarization behavior. The specific orientation and spatial distribution of these anisotropies remain unresolved, though Fast Fourier Transform (FFT)-supported analyses of AFM images^17^ may help clarify these patterns. Spatial correlation of polarimetric measurements with nanoscale data from AFM imaging in a manner analogous to previous comparisons with stained histology slides^1^ could reveal whether the observed microscale polarization signatures correspond to specific nanoscale fibrillar structures (e.g., D-banding periodicity), enabling a more informed analysis. In this way, MMP analysis may both guide the identification of regions of interest for nano-scale targeted analysis and provide a framework for linking collagen ultrastructure to measurable optical signatures, thereby offering deeper mechanistic insight into EDS subtype differences.

The current imbalance across groups (3 healthy controls, 5 cEDS, and 11 hEDS) and overall small sample size (n=19) reflects both the exploratory nature of this pilot study and the recruitment challenges inherent to rare diseases such as EDS.^1^ The samples used in this study emerged from a pilot study^1^ designed as an exploratory research project, prioritizing inclusivity over strict group parity to maximize data capture and hypothesis generation. With that said, expanding the dataset and balancing sample sizes across the two EDS groups will be essential for a more rigorous evaluation. Larger cohorts would enable clinically relevant analyses such as receiver operating characteristic (ROC) curves to assess diagnostic performance,^53^ and three-way comparisons (healthy, cEDS, hEDS) using ANOVA^54^ or Kruskal–Wallis,^55^ and multivariate approaches such as principal component analysis (PCA).^56^ The linear polarization parameter $P_{L}$ has already shown potential for distinguishing between healthy and EDS tissue, as well as between cEDS and hEDS, highlighting the value of extending analysis to all three groups. Incorporating clinical variables, such as age, race, and relevant medical history, will also be critical to determine whether these polarization patterns generalize across patient subgroups. In addition, machine learning approaches may help identify nonlinear combinations of polarimetric parameters that improve group separation beyond what is possible with individual biomarkers. Future studies with a larger sample size, informed by these findings, will thus incorporate stratified recruitment strategies and power calculations to address these limitations.

Despite these constraints, this exploratory study shows that label-free PLM can provide quantitative information on tissue microarchitecture without the need for staining or complex sample preparation. Compared with resource-intensive approaches such as TEM,^13^ it offers a more direct, affordable, accessible, and scalable method for assessing structural changes in EDS. The parameters obtained from patient skin provide objective quantitative measures of extracellular matrix reorganization and may capture features relevant to disease classification.^1^^,^^7^^,^^12^^,^^14^^,^^17^^,^^52^^,^^57^ Although preliminary, these findings indicate that polarimetric biomarkers could contribute complementary information to current diagnostic criteria,^28^ supporting future work on earlier detection and subtype classification.

## 4 Conclusion

EDS remains a diagnostically challenging condition, with current approaches lacking objective biomarkers. In this proof-of-concept study, PLM combined with quantitative analysis identified three polarization parameters ($P_{L}$, β, and ψ) that showed statistically significant differences between healthy skin and EDS-affected tissue. Furthermore, in the direct comparison between cEDS and hEDS, five parameters ($P_{L}$, $r_{L}$, $P_{1}$, $P_{3}$, and $P_{\mathrm{tms}}$) showed significant differences. As seen, $P_{L}$ biomarker demonstrated consistent discriminative capacity in both analyses. These results support the potential of polarimetric parameters derived via the simple PLM approach as quantitative biomarkers for EDS classification, underscoring the need for validation in larger, demographically balanced cohorts to assess robustness and clinical applicability.

## Supplementary Material

10.1117/1.BIOS.3.1.015002.s01

## Data Availability

The data that support the findings of this article are not publicly available. They can be requested from the author at k.tumanova@mail.utoronto.ca.
