# Supplementary material for: Label-free differentiation of classical and hypermobile Ehlers–Danlos syndromes using Mueller matrix polarimetry
Source: Biophotonics Discov. 2026 Jan 23;3(1):015002. doi: 10.1117/1.BIOS.3.1.015002 (PMC13052490; doi:10.1117/1.BIOS.3.1.015002)
Supplement: Supplementary file 1 [file BIOS_003_015002_SD001.pdf]

Supporting Information

Table S1 Summary of means (μ), medians and standard deviations (σ) across healthy, cEDS and hEDS samples, with *p*-values for healthy versus. pooled EDS and cEDS versus. hEDS comparisons, for parameters that showed statistical significance. The statistical analysis used, t-test (t) or Mann-Whitney-U (MWU), is indicated in parentheses. Statistically significant results are in bold.

Parameter		Healthy			p (Healthy versus. EDS)	cEDS			hEDS			p (cEDS versus. hEDS)
		μ	Median	σ		μ	Median	σ	μ	Median	σ	
1	P_L	6e-3	6e-3	1e-3	**0.003 (t)**	5e-3	5e-3	5e-4	5e-3	5e-3	3e-4	**0.03 (t)**
2	β	5e-3	5e-3	1e-3	**0.02 (t)**	3e-3	3e-3	5e-4	3e-3	3e-3	6e-4	0.2 (t)
3	ψ	-1e-5	-7e-7	1e-5	**0.048 (t)**	1e-6	9e-7	9e-7	2e-6	8e-7	5e-6	0.6 (MWU)
4	r_L	5e-2	4e-2	3e-3	0.9 (t)	4e-2	3e-2	9e-3	5e-2	5e-2	6e-3	**0.008 (t)**
5	P_1	-1e-2	-1e-2	7e-4	0.5 (t)	-6e-3	-7e-3	4e-3	-9.7e-3	-9e-3	3e-3	**0.046 (t)**
6	P_3	3e-2	3e-2	1e-3	0.9 (t)	3e-2	3e-2	6e-3	3e-2	3e-2	5e-3	**0.02 (t)**
7	P_{tms}	2e-2	2e-2	2e-3	0.9 (t)	2e-2	2e-2	5e-3	2e-2	2e-2	3e-3	**0.01 (t)**

Table S2 Summary of means (μ), medians and standard deviations (σ) across healthy, cEDS and hEDS samples, with *p*-values for healthy versus. pooled EDS and cEDS versus. hEDS comparisons, for parameters that did not reach statistical significance. The statistical analysis used, t-test (t) or Mann-Whitney-U (MWU), is indicated in parentheses.

Parameter		Healthy			p (Healthy versus. EDS)	cEDS			hEDS			p (cEDS versus. hEDS)
		μ	Median	σ		μ	Median	σ	μ	Median	σ	
1	Δ	9.8e-1	9.8e-1	4e-3	0.2 (t)	9.7e-1	9.7e-1	1e-2	9.7e-1	9.7e-1	3e-3	0.8 (t)
2	b	8e-1	8e-1	2e-2	0.2 (MWU)	7e-1	8e-1	1e-1	8e-1	8e-1	4e-2	1 (MWU)
3	P_C	4e-4	7e-4	6e-4	0.6 (MWU)	4e-4	3e-4	3e-4	3e-4	4e-4	5e-4	0.9 (MWU)
4	D	2e-2	1e-2	4e-3	0.3 (MWU)	1e-2	1e-2	2e-3	1e-2	1e-2	5e-3	0.3 (MWU)
5	D_C	-1e-2	-1e-2	4e-3	0.3 (t)	-3e-3	-6e-3	7e-3	-1e-2	-1e-2	7e-3	0.07 (MWU)
6	D_L	6e-3	6e-3	3e-4	0.4 (t)	6e-3	6e-3	6e-4	6e-3	6e-3	4e-4	0.7 (MWU)
7	t_1	6e-3	6e-3	8e-4	0.0503 (t)	5e-3	5e-3	4e-4	6e-3	6e-3	3e-4	0.1 (t)
8	A	2e-2	1e-2	2e-3	0.9 (MWU)	2e-2	2e-2	2e-3	2e-2	2e-2	5e-4	0.8 (t)
9	R	9e-1	9e-1	5e-2	0.8 (t)	1	1	6e-1	1	1	3e-1	0.8 (t)
10	δ	2	2	8e-2	0.2 (t)	2	2	4e-1	2	2	2e-1	0.06 (t)
11	q_L	1e-2	1e-2	9e-4	0.8 (t)	1e-2	1e-2	3e-3	1e-2	2e-2	2e-3	0.07 (t)

12	P_2	7e-3	5e-3	3e-3	0.8 (MWU)	3e-4	-2e-4	6e-3	8e-3	6e-3	7e-3	0.07 (t)
13	P_4	-9e-5	-6e-5	5e-5	0.9 (MWU)	-2e-5	-6e-6	8e-5	-2e-4	-9e-5	2e-4	0.1 (t)
14	P_5	-2e-4	1e-4	7e-4	0.9 (MWU)	-9e-5	-1e-4	3e-4	3e-4	3e-4	7e-4	0.8 (t)
15	P_6	6e-4	2e-3	2e-3	0.7 (MWU)	1e-3	1e-3	5e-4	1e-3	1e-3	4e-4	1.0 (MWU)
16	P_7	3e-3	3e-3	2e-4	0.06 (MWU)	3e-3	3e-3	3e-4	3e-3	3e-3	3e-4	0.4 (MWU)
17	P_8	1e-6	1e-6	1e-6	0.9 (t)	5e-7	4e-7	5e-7	9.7e-7	9.8e-7	1e-6	0.4 (t)

16
